# Treatment of a high-energy transsyndesmotic ankle fracture: A case report of “logsplitter injury”

**DOI:** 10.1097/MD.0000000000019380

**Published:** 2020-02-28

**Authors:** Zhaowei Yin, Zitao Wang, Dawei Ge, Junwei Yan, Chunzhi Jiang, Bin Liang

**Affiliations:** Department of Orthopaedic, Nanjing First Hospital, Nanjing Medical University, Nanjing, PR China.

**Keywords:** ankle fracture, deltoid ligament, logsplitter injury, syndesmotic injury, syndesmotic screw

## Abstract

**Introduction:**

The “logsplitter injury” is a special type of ankle fractures that results from high energy violence with combined rotational forces and axial loads. So far, the diagnose and treatment of “logsplitter injury” remain largely unsettled and related literature is rare.

**Patient concerns:**

An 18-year-old male fell from a fence and got his left ankle injured with severe malformation and swollen condition. No open wound was observed.

**Diagnosis:**

Logsplitter injury, ankle fracture (AO/OTA classification 44C1.1, Lauge-Hansen classification: pronation-external rotation).

**Interventions and outcomes:**

The patient was initially treated by internal fixation of fibular, repair of deltoid ligaments, and 1 syndesmotic screw fixation. When the X-ray applied after surgery, another 2 syndesmotic screws were performed to enhance stability. The syndesmotic screws were removed at 12-week and 16-week respectively. The patient was allowed for full weight-bearing immediately. However, the syndesmotic space was slightly increased compared to the contralateral side in CT views at 1-year follow-up, the function outcome was satisfied.

**Conclusion:**

The logsplitter injury is a high-energy ankle fracture that requires both axial and rotational load. It is categorized as 44B or 44C by the AO/OTA classification. In the classification scheme of Lauge-Hansen, our case is in line with the pronation-external rotation classification. Anatomic reduction and fixation of ankle syndesmotic injuries are required to restore the biomechanics of the ankle joint so that long-term complications can be prevented. How to fixation the syndesmosis, whether to reconstruct the deltoid ligament remains in debate in the treatment of logsplitter injury, whether and when to remove the syndesmotic screws were still debated. Correct surgical intervention is successful in the treatment of “logsplitter injury”, however, the optimal fixation of syndesmosis and repair of deltoid ligaments need further investigate.

## 1 Introduction

Ankle fracture is among the most frequently encountered intraarticular fractures worldwide.^[1]^ During fractures and dislocations of the ankle joint, distal tibiofibular syndesmotic disruption occurs at a rate of approximately 10% to 20%,^[2,3]^ usually resulting from external rotation of the talus in the ankle mortise.^[4]^ However, during high energy injuries, especially combined with a vertical axial load, patients can present with the talus wedged into the distal tibiofibular joint, resulting in a trans-syndesmotic ankle dislocation with multiple ligaments compromised. This mechanism is further described as a “logsplitter injury”, as it is similar to that of a logsplitter wedge for splitting wood.^[5]^ The injury pattern comprises syndesmotic avulsion, ankle dislocation, combined tibial plafond fracture and soft tissue injury, making the logsplitter injury a considerable challenge for trauma surgeons.^[6]^

Due to the rarity in both clinical cases and the literature, treatment of logsplitter injury and the outcome prognosis remains unclear, leaving much to be considered.^[7]^ It is well recognized that the logsplitter injury requires emergency reduction and surgical procedures, if permitted, for its apparently complicated injury pattern.^[5,6,8]^ In this case, we report and discuss the diagnosis, treatment and outcomes of a patient with a logsplitter injury pattern who was admitted to our hospital. Permission from the patient was obtained for reporting this case.

## 2 Case report

**Figure 1:**
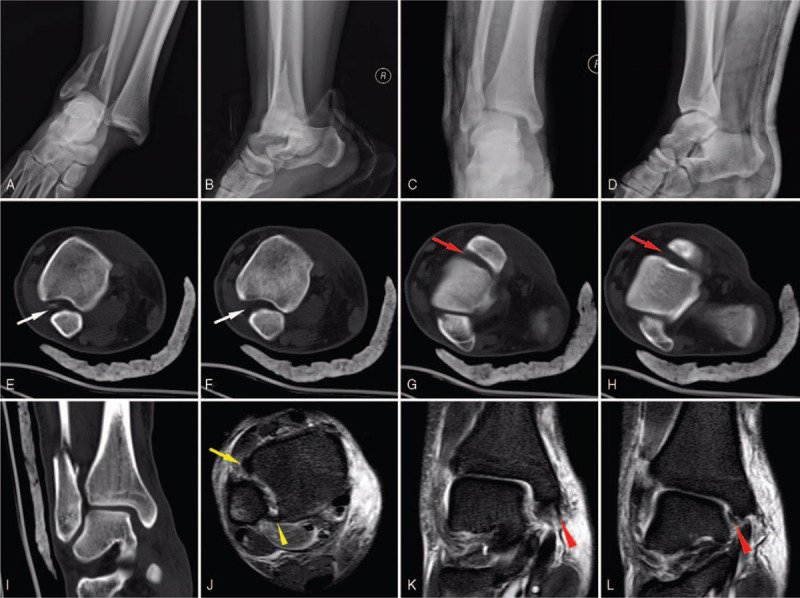
The preoperative imaging materials. (A,B) Anteroposterior and lateral X-ray image of injured ankle immediately. (C,D) Anteroposterior and lateral X-ray image of injured ankle after manual reduction and cast immobilization. (E–H) The different cross sections of CT. The anterior distal tibiofibular syndesmosis (red arrow) and medial ankle mortise (white arrow) were obviously increased. (I) Coronal section of CT shows the asymmetric mortise. (J–L) The MRI views of injured ankle. The anterior-inferior tibiofibular ligament (AITFL) was ruptured (yellow arrow), while the posterior-inferior tibiofibular ligament (PITFL) was intact (yellow triangle arrow). The superior and deep deltoid ligament were also ruptured (red triangle arrow).

An 18-year-old male patient was admitted to the emergency department after he fell to the ground while climbing a 2-m fence. The weight and height of the patient was 101 kg and 1.76 m, respectively, and his body mass index (BMI) was 32.6 kg/m^2^. This patient was clear minded by presentation. He was unable to bear weight on his right ankle, which was in a severely malformed and swollen condition, but there appeared to be no open wound on the skin. The patient complained of pain and swelling and was unable to perform active ankle movements. Meanwhile, passive ankle movements were limited and painful. No other discomfort or past medical history was declared. A normal finding was obtained on neurovascular examination. A direct radiographic examination of the ankle showed a displaced lateral malleolar fracture with the talus wedged into the distal tibiofibular joint (Fig. 1 A-B). An immediate manual reduction followed by fixation with plaster was performed by orthopedists (Fig. 1 C-D). To further understand the pattern of injury, a computerized tomography (CT) examination with 3D reconstruction was performed, which demonstrated distal fibular fracture and widening of the distal tibiofibular syndesmosis (Fig. 1 E-H). Magnetic resonance imaging (MRI) revealed rupture of the superior and deep deltoid ligament and the anterior-inferior tibiofibular ligament (AITFL) (Fig. 1 J-L). Based on the radiological outcomes, the fracture-dislocation classification was considered as 44C1.1 (AO/OTA classification) and pronation-external rotation (Lauge-Hansen classification). Due to the extreme ankle instability caused by bone and ligament injury, surgical intervention was recommended and performed after 7 days until swelling in the foot and ankle had adequately dissipated, as indicated by a positive wrinkle test.

**Figure 2:**
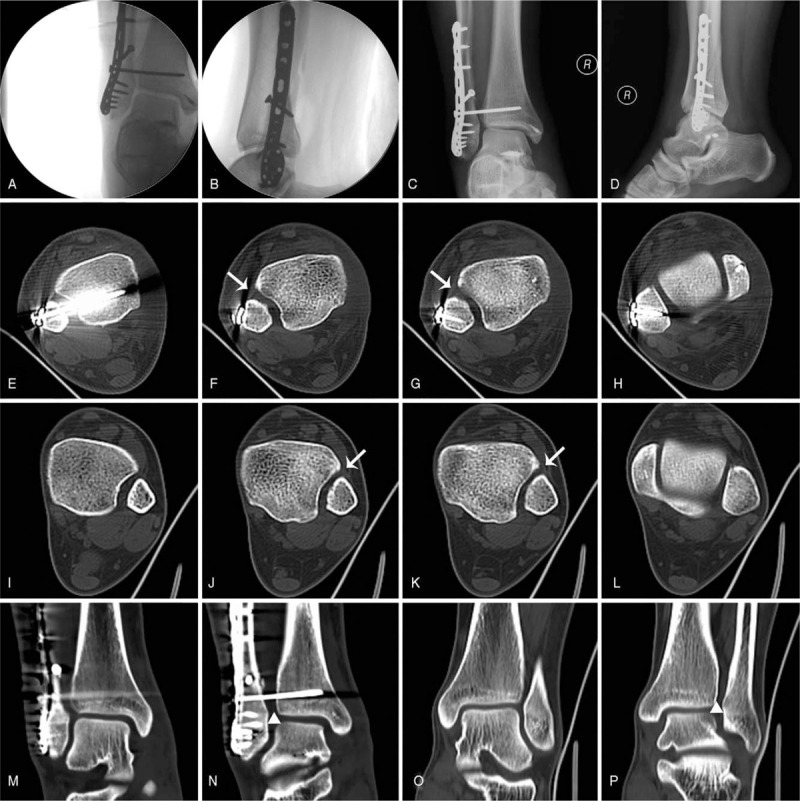
(A,B) The intraoperative fluoroscopy view. (C,D) Anteroposterior and lateral X-ray image 1-week post-operation. The distal tibiofibular syndesmosis was wider than intraoperation, notice the syndesmotic screw had a tendency to loosen. (E–L) The cross sections of CT views of bilateral side. The syndesmosis (white arrow) and mortise space (H,L) of injured side were slight wider than contralateral side. (M–P) The coronal sections of CT views of bilateral side. The syndesmosis (white triangle) and mortise space (M,O) of injured side were slight wider than contralateral side.

For the surgery, the patient was placed in a supine position under spinal anesthesia and tourniquet control. First, a curved 6 cm incision was made just below the medial malleolus and extended distally to expose the ruptured deltoid ligament and its individual components. Two suture anchors were placed at the insertion point of the deep component of the deltoid ligament of the talus, and 1 suture anchor was placed into the tip of anterior colliculus of the medial malleolus in preparation for deltoid ligament reconstruction. After that, an incision of approximately 10 cm was made on the lateral side of the fibula. Soft tissue was incised by sharp dissection to expose the broken site of the fractured fibula. Reduction of the fibula was conducted to recover the length, followed by applying a screw vertical to the fracture line for stabilization. Then, a 3.5 mm locking compression plate (LCP) was placed for neutralization. A large reduction clamp was maintained at the space of the syndesmosis and medial malleolus while the ankle joint was held in a neutral position. The mortise width and distal tibial-fibular space were restored and confirmed under C-arm fluoroscopy, and a 3.5 mm syndesmotic screw was applied. Finally, we repaired the deltoid ligaments with sutures (Fig. 2 A-B).

**Figure 3:**
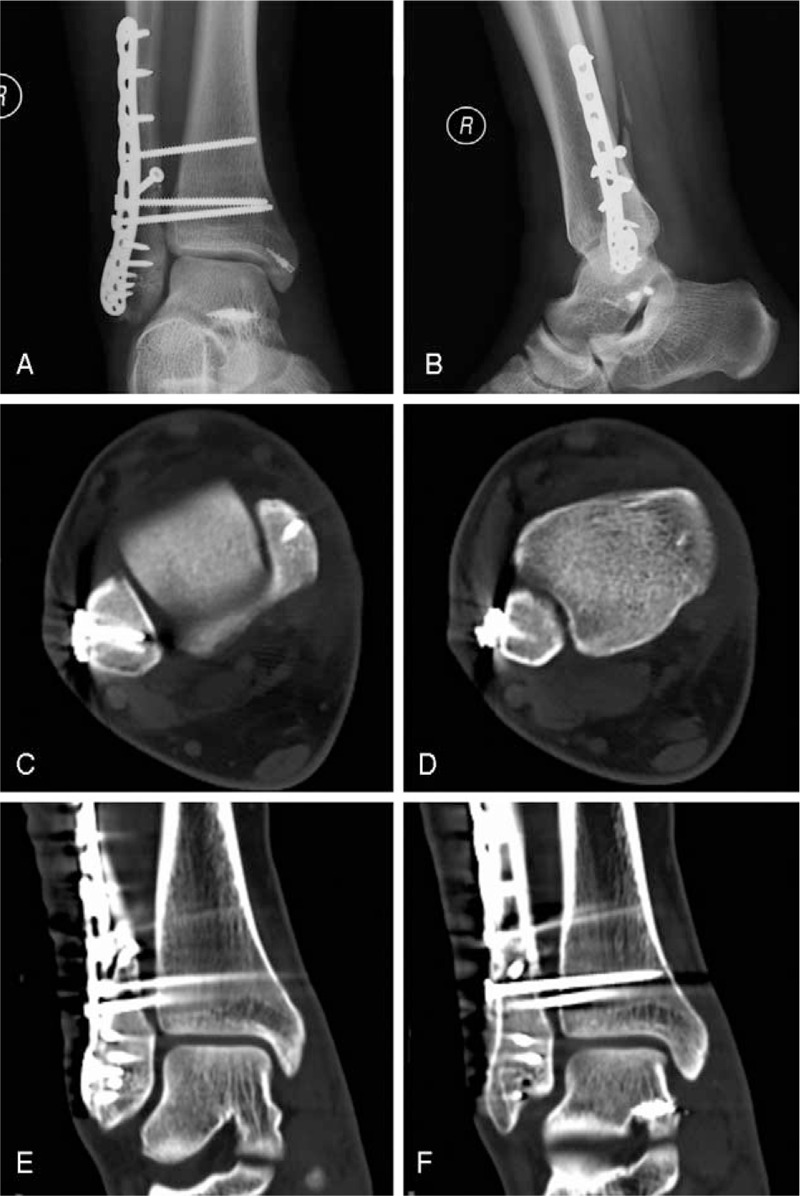
(A,B) Anteroposterior and lateral X-ray image re-operation. (C–F) post-operative CT views shows the syndesmosis and mortise almost normal.

Plaster was not applied after surgery. We applied early functional exercise, to include passive motion of the ankle and mobilization of the toes and knee joint 2 days postoperatively. Weight bearing was avoided. However, a radiographic examination 1 week after surgery showed that the distal tibiofibular syndesmosis was slightly wider than on intraoperative fluoroscopy, as the syndesmotic screw has a tendency to loosen (Fig. 2 C-P). Therefore, a re-operation was conducted to deal with the syndesmosis. Using the same lateral approach as the first operation, a Weber forceps was used to reduce the gap between the tibia and fibula. Then, 2 more syndesmotic screws were inserted outside of the plate. The former syndesmotic screw was substituted with a longer one. Stability of the distal tibiofibular syndesmosis was confirmed by the “HOOK” test (Fig. 3).

**Figure 4:**
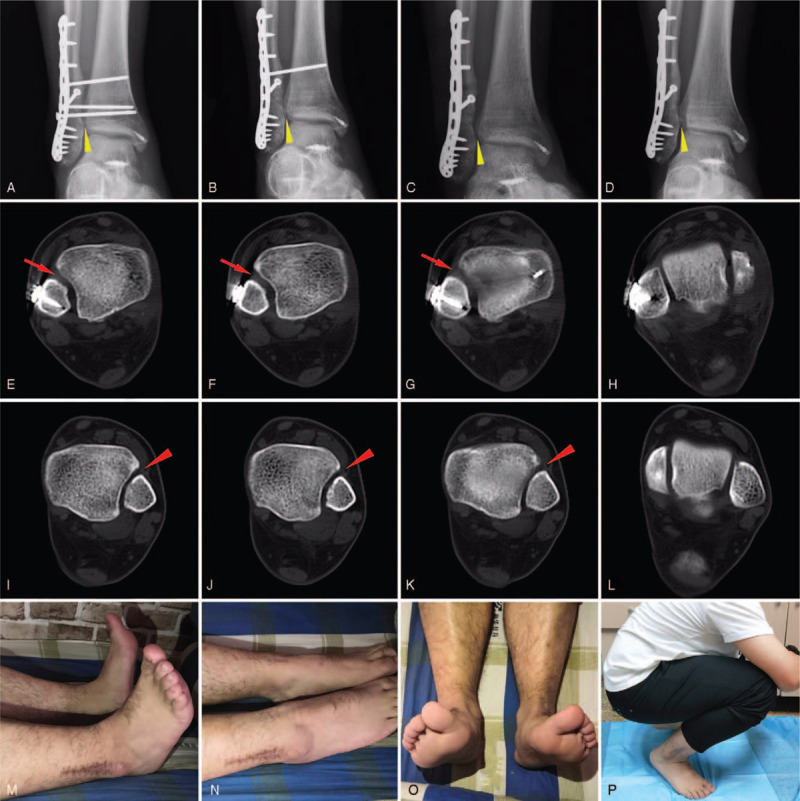
(A) 12-week post-operative X-ray image, (B) X-ray image after 2 syndesmosis screws were removed at 12-week. (C) X-ray image after all syndesmosis screws were removed at 16-week. (D) X-ray image at 1-year follow-up. After all screws removed, the syndesmosis was slightly increased (yellow triangle arrows). (E–L) the CT images at 1-year follow-up confirmed the increase of injured syndesmosis (red arrows) than contralateral side (red triangle arrows). (M–P) The functional outcomes of injured ankle at 1-year follow-up showed satisfied result, the ROM of dorsiflexion and plantarflexion was almost normal.

Early passive motion of the ankle was continuously performed by a physical therapist. The radiographic examination and CT after the second operation showed a well reduced and aligned distal tibiofibular syndesmosis. Twelve weeks postoperatively, 2 syndesmotic screws were removed, and partial weightbearing was allowed with the assist of single crutch; 16 weeks postoperatively, the last syndesmotic screw was removed. Full weight bearing was allowed immediately. At 1 year after surgery, the patient was called back to the hospital for a radiographic and clinical examination. Radiographs and CT showed union of the fibular fracture; however, the syndesmotic space was slightly increased compared to the contralateral side (Fig. 4). Fortunately, the patient was free of pain and returned to his previous level of daily activity; the range of motion of the injured ankle was nearly parallel with the contralateral side (Fig. 4). According to American Orthopedic Foot and Ankle Society Score (AOFAS),^[9]^ the score was 90. This study was approved by the ethics committee of Nanjing first hospital. Signed written informed consents were obtained from the patient.

## 3 Discussion

The logsplitter injury is a high-energy ankle fracture that requires both axial and rotational loads.^[5]^ These fractures are categorized as 44B or 44C by the AO/OTA classification.^[10]^ Wang et al suggested a typical and untypical logsplitter injury classification according to the injury mechanism and the degree of the talus wedged into the distal tibiofibular joint.^[6]^ Most trauma patterns result from high energy events such as a car accident or falling from a height. In the classification scheme of Lauge-Hansen,^[11]^ ankle fracture dislocations accompanied with syndesmotic disruption can be divided into supination-external rotation (SER), pronation-abduction (PAB), pronation-external rotation (PER).^[5]^ Forced dorsiflexion of foot combined with lateral rotation and pronation of ankle may cause inferior tibiofibular syndesmotic disruption and talus upward dislocation.^[12]^ In this case, the patient suffered from an axil violence by fell from a 2-m high fence. According to the radiological examinations, the deltoid ligament and AITFL ruptured, the fibular fracture line was spiral, the posterior inferior tibiofibular ligament (PITFL) was intact, these signs indicated that the foot was in the pronation position at the time of injury, and suffered a combined force of external rotation and axial direction. This is consistent with a typical injury pattern.

The complications of ankle fractures especially high energy transyndesmotic injuries were not uncommon. These complications may be classified as perioperative, early postoperative, late postoperative,^[13]^ include wound complications and surgical site infection, malreduction, loss of reduction and post-traumatic osteoarthritis, nonunion, neurologic complications, thromboembolic complications.^[14]^

Anatomic reduction and fixation of ankle syndesmotic injuries are required to restore the biomechanics of the ankle joint so that long-term complications can be prevented.^[15]^ The syndesmotic ligament complex stabilizes the fibula to the tibia. There are 4 major ligaments that compose the ankle syndesmosis; they are the anterior inferior tibiofibular ligament (AITFL), the posterior inferior tibiofibular ligament (PITFL), the inferior transverse tibiofibular ligament and the interosseous ligament.^[16]^ Avulsion of the previously described ligaments can be diagnosed by radiographic signs.^[17]^ Clinically, 3.5 mm screws are widely used for syndesmotic fixation. However, the number of screws to use for logsplitter injury remains unclear. Wang et al suggested that a single syndesmotic screw placed 3 to 4 cm above the syndesmosis could maintain stability in an untypical type fracture.^[6]^ Van Zuuren recommends that multiple screw placement should be considered only for instability in obese patients, which requires extra support.^[2]^ In this case, due to the relatively high energy violence and excess body weight of our patient, 2 or 3 screws holding at least 3 layers of cortex were required for syndesmotic fixation. Additionally, 4.5 mm screws can be considered. However, the distal fibular LCP applied in this patient did not allow any 4.5 mm screws to be placed due to the diameter of its holes. Thus, we first placed a 3.5 mm screw through the elliptic hole. Unfortunately, the screw did not have enough holding force. In the second operation, we had to apply 2 syndesmotic screws outside the plate, which carried a risk of iatrogenic fracture of the fibula.

Of note, the deltoid ligament complex contributes to restraint against valgus tilting of the talus.^[18]^ Whether to reconstruct the deltoid ligament remains in debate in the treatment of logsplitter injury. Previous research found that repair of the deltoid ligament is necessary only when the reduction of the lateral malleolus is guaranteed to reduce the talus within the mortise.^[19]^ However, recent biomechanism research revealed that deltoid repair has similar outcomes for both lateral and medial drawer reduction with syndesmosis fixation, but these measures can only reach normal values when both are repaired.^[20]^ Of course, the ligament may heal itself without surgical repair, but biomechanical function can hardly be restored.^[21]^ A retrospective study showed that despite the similar clinical outcomes with or without deltoid ligament repair, better results were obtained when the deltoid ligament was repaired in patients with syndesmotic fixation.^[22]^ In our case, the patient was quite young and strong, which required strong ankle stability to handle his future high activity level. Meanwhile, after the screws were fixed, a valgus and external rotation stress test was performed on the bilateral side, and the medial malleolus space of the injured side was increased. Therefore, we still performed the deltoid ligament repair. These are well accepted indications for deltoid ligament repair.^[23]^

Whether to remove the syndesmotic screws or not remains under debate. Manjoo et al^[24]^ suggested favorable outcomes with screw removal. However, most research^[8,25–28]^ so far has revealed no significant functional, clinical or radiological differences between the 2 groups (removed and retained screws), although there is evidence that rigid syndesmotic fixation has a reverse impact on physiological tibiofibular movement and dorsiflexion.^[29]^ In our case, in order to minimize the incidence of breakage of the screws and restore the normal movement between the distal tibia and fibula, we removed the screws. However, the postoperative CT showed a slight widening of the tibiofibular space compared with the preoperative images. Fortunately, no detrimental functional effect was found. Reports from previous studies are in accordance with ours.^[30,31]^

The time to start weight bearing and the removal of syndesmotic screws was carefully considered for the patient during postoperative management. Previous studies have suggested that weight bearing can be allowed 2 weeks after the operation, while screw removal can be considered after 6 months.^[23]^ Considering the age and activity level of the patient, the removal process was decided individually. More effort should be made to further investigate the rehabilitation process for the logsplitter injury patient. In conclusion, several learning points from this patient experience may be obtained. First, patients who are strong or obese require at least 2 syndesmotic screws with at least 3 cortices holding each screw. Second, repair of the medial collateral ligament is highly recommended in young and energetic patients, who usually participate in various sports activities. Last but not least, although the functional outcome of this patient was satisfactory, the tibial-fibular space slightly increased after the syndesmotic screws were removed, which led us to question the necessity of the screw removal.

## Author contributions

**Conceptualization:** Zhaowei Yin.

**Project administration:** Chunzhi Jiang.

**Resources:** Junwei Yan.

**Writing – original draft:** Zhaowei Yin, Zitao Wang.

**Writing – review & editing:** Bin Liang.
